# Is over recruitment to a single site problematic for surgical trials?

**DOI:** 10.1186/1745-6215-16-S2-O48

**Published:** 2015-11-16

**Authors:** David Beard, Andrew Price, David Murray, Loretta Davies, Cushla Cooper, Jonathan Cook

**Affiliations:** 1 University of Oxford, Oxford, UK

## 

Most trials have one site (or more) which excels at recruitment and adds patients at a greater rate than others. In a reasonable attempt to complete recruitment into a trial these sites are often encouraged to continue adding patients despite generating a disproportionate number of patients. This study uses the TOPKAT data to demonstrate the potential danger of this frequent practice.

TOPKAT is a multi-centre NIHR HTA sponsored surgical trial of over 500 patients designed to compare the cost efficacy of partial versus total knee replacement in the NHS using the Oxford Knee Score as primary variable. One year follow up results are now available on 531 patients.

A single centre recruited 107 out of a total of 531 patients. Despite successful randomisation and balance between groups the baseline demographics and self-reported data was different in this single centre compared to that for the entire study sample. Furthermore, the differences found in the primary outcome for the main study at 1 year were not reflected in the sub analysis of the single site. Had the conclusions been based on the data from the single site, an entirely different interpretation for the study results would have been appropriate.

The difference in the conclusion obtained from the two different data sets cannot be dismissed because of the high proportion of patients (20%) from the single centre. The work highlights the perils of allowing a single centre to over recruit.

